# Parity History Determines a Systemic Inflammatory Response to Spread of Ovarian Cancer in Naturally Aged Mice

**DOI:** 10.14336/AD.2017.0110

**Published:** 2017-10-01

**Authors:** Ulises Urzua, Carlos Chacon, Luis Lizama, Sebastián Sarmiento, Pía Villalobos, Belén Kroxato, Katherine Marcelain, María-Julieta Gonzalez

**Affiliations:** ^1^Laboratorio de Genómica Aplicada, Facultad de Medicina, Universidad de Chile; ^2^Programa de Virología, ICBM; ^3^Programa de Genética Humana, ICBM; ^4^Programa de Biología Celular y Molecular, ICBM; ^5^Departamento de Oncología Básica y Clínica, Facultad de Medicina, Universidad de Chile, Santiago, Chile

**Keywords:** age, parity, ovarian cancer, menopause, cytokine, inflammation, mouse model

## Abstract

Aging intersects with reproductive senescence in women by promoting a systemic low-grade chronic inflammation that predisposes women to several diseases including ovarian cancer (OC). OC risk at menopause is significantly modified by parity records during prior fertile life. To date, the combined effects of age and parity on the systemic inflammation markers that are particularly relevant to OC initiation and progression at menopause remain largely unknown. Herein, we profiled a panel of circulating cytokines in multiparous versus virgin C57BL/6 female mice at peri-estropausal age and investigated how cytokine levels were modulated by intraperitoneal tumor induction in a syngeneic immunocompetent OC mouse model. Serum FSH, LH and TSH levels increased with age in both groups while prolactin (PRL) was lower in multiparous respect to virgin mice, a finding previously observed in parous women. Serum CCL2, IL-10, IL-5, IL-4, TNF-α, IL1-β and IL-12p70 levels increased with age irrespective of parity status, but were specifically reduced following OC tumor induction only in multiparous mice. Animals developed hemorrhagic ascites and tumor implants in the omental fat band and other intraperitoneal organs by 12 weeks after induction, with multiparous mice showing a significantly extended survival. We conclude that previous parity history counteracts aging-associated systemic inflammation possibly by reducing the immunosuppression that typically allows tumor spread. Results suggest a partial impairment of the M2 shift in tumor-associated macrophages as well as decreased stimulation of regulatory B-cells in aged mice. This long term, tumor-concurrent effect of parity on inflammation markers at menopause would be a contributing factor leading to decreased OC risk.

Natural aging is concomitant with a systemic low-grade chronic inflammation that contributes to age-related morbidity and mortality [1]. It is currently well accepted that tissue and organ impairment observed with aging is caused in large part by accumulation of various forms of molecular and cellular damage including genomic instability, epigenetic changes, altered proteostasis, dysregulated nutrient sensing, deficient mitochondrial function, stem cell depletion and altered intercellular communication [2].

Unique to women, menopausal hormone imbalances occurring as a consequence of ovarian decline, seem to contribute to an altered systemic inflammatory status [3-5], resulting in an increased susceptibility to several chronic pathologies such as osteoporosis, metabolic, cardiovascular and neurodegenerative diseases as well as -relevant to this study- various types of cancers [6]. The peri- and post-menopausal stages in women are characterized by elevated circulating serum levels of several inflammation-related cytokines including TNF-α, IL-6, IL-1β, IL-8, MCP-1, RANTES, MIP-1α, IL-2, GM-CSF, G-CSF, IL-4, IL-10, IL-12 and IL-18 [3, 7-14], which acting in combination, could lead to the observed low-grade systemic inflammatory state.

Ovarian cancer (OC) incidence and mortality significantly increases with age, both parameters reaching a peak during post-menopause. Elevations of some of the above mentioned cytokines have been shown a direct correlation with increased OC risk [15-18], while others have been linked to reproductive hormones known to be altered during the menopausal transition. The gonadotropins follicle stimulating hormone (FSH) and luteinizing hormone (LH) typically increase at menopause due to impairment of the hypothalamic-pituitary-gonadal (HPG) axis and, importantly, have been proposed to play a role in OC pathogenesis [19]. Extending this link, plasma levels of the pro-inflammatory cytokines IL-1β, IL-6 and TNF-α were positively correlated with circulating FSH in premenopausal women [5]. Similarly, the C-C motif chemokine ligand 2 (CCL2/MCP-1) was positively correlated with FSH levels during the early and late menopausal transition [14]. In female rheumatoid arthritis patients, short-term fluctuations of both FSH and LH were positively associated with changes in levels of TNF-α, IL-1β and other pro-inflammatory cytokines [20].

A major contributing factor to the roles of menopausal hormone imbalances and the direct effect of certain cytokines on OC risk and progression in post-menopausal women, is their reproductive history during precedent fertile phase [21]. One or more full-term pregnancies significantly reduces OC risk, while nulliparity increases the risk [22]. Since an equivalent extent of OC risk reduction is achieved by progestin-based oral contraceptives, protection could be the result of a reduced number of ovulatory cycles concomitant with increased progesterone (P4) or progestin exposures [23]. Thus, uninterrupted ovulation might play a role in OC origin through cumulative DNA damage incurred during the repetitive cycles of wound rupture-repair of the ovarian surface epithelium [24] while the preventive actions of P4 (or progestin) could be mediated by an apoptotic effect [25] or induction of senescence on transformed ovarian surface cells [26]. Whatever the OC pro- or anti-oncogenic mechanisms are, they have to be long-term, i.e. they should develop and persist during cell and tissue aging.

**Figure 1.**
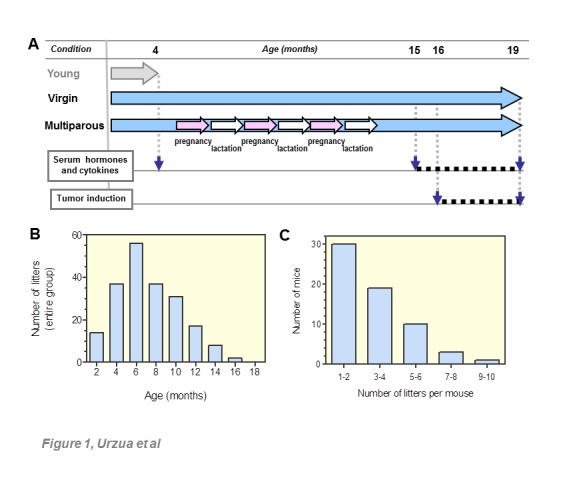
Study design and parity records **(A)** Two groups of C57BL6 female mice (n=70 per group) were maintained from 3-20 months old in virgin (nulliparous) or multiparous conditions. A smaller third group, composed of young adult 4 months old virgin mice (n=6), was used as reference controls in some assays. Multiparous mice were allowed to breastfeed their pups until 21-days old. Circulating cytokines and hormones were measured once per month in 3-4 distinct, randomly taken mice aged 15-19 months old. Tumor induction was initiated at 16 months old (n=8 per condition) with cytokines and hormones measured similarly. The age scale shown is not proportional. See Methods section for additional details. **(B)** Chart corresponds to the total number of litters as a function of age. **(C)** depicts the distribution of number of litters per individual mice over the reproductive period, both data-sets for the entire multiparous group.

With the goal of investigating how parity modulates chronic, age-related systemic inflammation and how that modulation contributes to OC risk at menopause, we herein describe a profile of circulating hormones and cytokines in multiparous versus virgin C57BL/6 female mice subjected to tumor induction at peri-estropausal age. From over 30 cytokines evaluated, seven increased with age regardless of parity status. The same seven cytokines decreased only in multiparous mice as a response to tumor induction with transformed OC syngeneic cells. Both virgin and multiparous mice developed intraperitoneal tumors and ascitic fluid, but multiparous animals showed an extended survival. Our results suggest that parity supports and extends the host immune response against intraperitoneal OC tumor spread during menopause.

## MATERIALS AND METHODS

### Animals and samples collection

Figure 1A summarizes the experimental protocol of this work. Wild type C57BL/6 mice were housed at *Bioterio Central* of *Facultad de Medicina*, *Universidad de Chile*. The animal study, protocol Nº 0536, was approved by the institution´s *Comité de Bioetica*. Wild type C57BL/6 mice were maintained under 12/12 hours’ light/dark schedule with regular food and water *ad libitum*. Virgin adult female mice, 12 weeks old, were randomly assigned to two experimental groups, virgin and multiparous (n=70 per group). Breeding trios were used for the multiparous mice; the trio included a fertile adult male mouse of proven fertility. Virgin mice were housed three females per cage in the absence of a male. Separate cages with virgin and with multiparous mice were maintained in close proximity, sharing the same rack to preserve estrous cycling in the virgin animals [27]. To evaluate reproductive aging, the length of estrous cycles was measured in the virgin group. Estrous cycling was addressed by fresh vaginal cytology at 10:00 AM during 15 consecutive days in random subsets of six virgin mice at 4, 15 and 18 months of age [28]. Pups of multiparous mice were weaned at 21-days old. Diestrous stage blood samples from four randomly chosen mice of each group, were collected monthly by submandibular bleeding [29]. The blood collection period for the intact, untreated mice group was between 15-19 months of age, and for the tumor-induced mice was between 16-19 months of age. Blood was incubated at room temperature for 30 min and centrifuged at 1000g for 5 min. Supernatant sera was carefully pipetted and stored at -20ºC in 25 μL aliquots for further analysis. Control blood samples were collected and processed similarly from 4 months old female mice at diestrous stage of cycle.

### Tumor induction in a syngeneic mouse model

With the goal of evaluating parity-dependent tumor progression, spontaneously-transformed mouse ovarian surface epithelial (MOSE) cells were injected in the peritoneal cavity of aged mice of both conditions. The IG-10 clonal line of MOSE cells was kindly provided by Dr Katherine Roby (UKMC, Kansas). Cells were cultured in DMEM supplemented with ITS (Sigma-Aldrich, MO) under 5% CO_2_ atmosphere [30]. When cultures reached 90% confluency, cells were released with trypsin, centrifuged at low speed and suspended in a minimal volume of HBSS solution (Sigma-Aldrich, MO). An aliquot was diluted and counted with a hemocytometer. A suspension volume containing 5×10^6^ cells was injected intraperitoneally with a tuberculin syringe to 16 months-old multiparous (n=8) and virgin (n=7) mice. One month earlier, males were removed from the multiparous mice cages. A set of control mice (n=4) was injected with HBSS vehicle solution. Animals were monitored once per week after injection. Sera samples were obtained from all animals two weeks after the injection and then monthly until death. Upon initial signs of ascitic fluid accumulation evaluated by abdominal enlargement, mice were supervised daily. Euthanasia by cervical dislocation was conducted according to signs of stress and affliction described by Morton and Griffiths [31]. Immediately upon death, blood samples and ascitic fluid were collected and processed for further analysis. Volumes of hemorrhagic ascitic fluid were measured (range 2-9 mL), clarified by centrifugation at 1,000g for 10 min, and stored at -20ºC in 0.5 mL aliquots. Intraperitoneal tumor loads were graded and scored as described by Roby *et al* [30].

### Circulating pituitary hormones and cytokines

Hormones and cytokines were quantified in serum using multiplex, magnetic bead-based Luminex® xMAP® assays manufactured by Milliplex® (Merck, USA). Specifically, the serum pituitary hormones FSH, LH, TSH and prolactin (PRL) were measured with the MPTMAG-49K panel and cytokines CCL2, IL-10, IL-5, IL-4, TNF-a, IL-1b, IL-12p70 and CXCL10 were quantified with the MCYTMAG-70K-PX32 panel. Assay methods were followed as indicated by the supplier. Prior to each assay, test samples were randomized and their identities were blind to the operators. In brief, serum samples were either used directly (10 μL; MPTMAG-49K kit) or diluted with an equal volume of assay buffer (25μL; MCYTMAG-70K-PX32 kit). Samples were loaded to the plate along with controls and standards provided with the kits. Then, 25 μL of the Pre-mixed Beads mixture were added to each well, the plate was sealed, covered with foil and incubated overnight in a shaker at 4°C. The entire content of wells was gently discarded. Plate was washed twice, 25-50 μL of Detection Antibodies solution were added and incubated 1 hr at room temperature with shaking. Then, 25-50 μL of Streptavidin-Phycoerythrin solution were added and incubated further 30 min at room temperature. The entire content of wells was gently discarded and washed twice. Finally, 100-150 μL of Sheath Fluid were added to each well and beads resuspended by shaking for 5 min. Median fluorescence intensity (MFI) of beads was read in a Luminex 200™ instrument (Luminex Corp., USA) operated through the xPONENT 3.1 software. Data processing was conducted with the Milliplex Analyst 3.5.5.0 software (Merck, USA) using the 5-parameter logistic curve.

### Statistics

Hormone and cytokine results are plotted as mean ± standard error of the mean. Since data was not distributed normally, the Kruskal-Wallis test was used to compare non-paired data among young and aged groups. The Dunn’s post test was applied for comparisons among these 3 conditions. The two tumor-induced, aged groups were separately compared with a Mann-Whitney U test. Significance was set at *p*<0.05. Analysis was performed with the GraphPad Prism 5.0 software.

## RESULTS

### Parity records, estrous cycling and pituitary hormone levels

**Figure 2.**
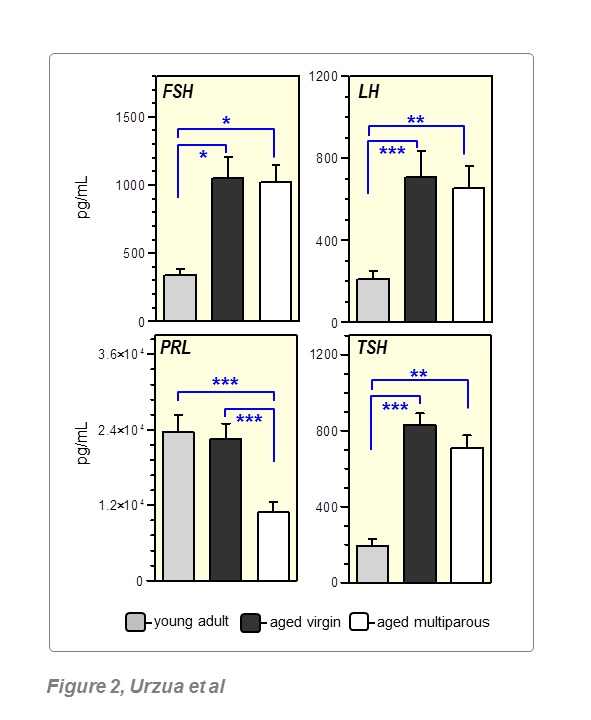
Circulating pituitary hormones in aged mice Serum levels of the indicated hormones were measured monthly in 4 randomly chosen female mice 15-19 months old. The reference group, was 4 months old young adults (n=6) was. Bars represent mean with SEM corresponding to the above-mentioned time period. (*) *p*<0.05, (**) *p*<0.01, (***) *p*<0.005, (§) 0.05<*p*<0.10. Further details in Methods.

For the parous group composed of 70 mice, a total of 202 litters were recorded. Seven (7) mice never had litters and were thus excluded from the study. This resulted in a mean of 3.2 litters per mouse. As shown in the histogram of Figure 1B, the maximum reproductive yield was observed at 6 months of age, and 95% of the litters were delivered before the age of 12 months. The number of litters, as a function of age, was consistent with a Gaussian distribution as indicated by three normality tests (not shown). Individual reproductive rates showed that 47.6% of mice had 1-2 litters, while another 46.0% had 3-6 litters (Fig. 1C). The mean length of estrous cycles increased from 4.6 days at four months of age, to 9.7 days in 15-18 months-old mice, a result that indicated reproductive senescence in the animals to be studied. The mean serum levels of FSH, LH, TSH and prolactin (PRL) in mice aged 15-19 months are shown in Figure 2. FSH, LH and TSH were significantly increased in aged versus young mice, regardless of parity history. PRL levels were unchanged between young and aged virgin mice, but were significantly lower in the aged multiparous group.

### Parity-dependent cytokine profiles

**Figure 3.**
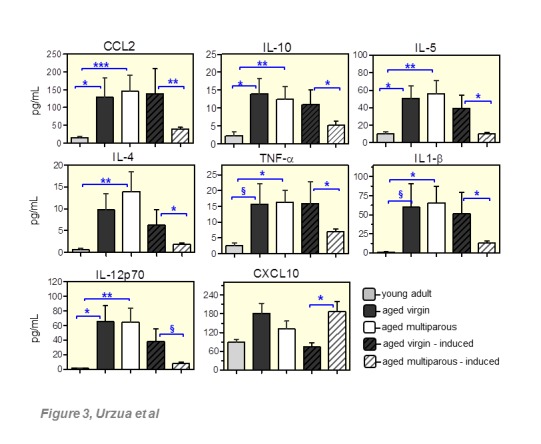
Circulating cytokines in control and tumor-induced aged mice Serum levels of the indicated cytokines were measured monthly from 15-19 months old in 3-4 randomly chosen female mice per group. Cytokines in the tumor-induced conditions (dashed bars, n=8 per group) were measured from the time of injection (16 months old) until time of death (19-19.5 months old). The young adult reference group (n=6) was 4 months old. Bars represent the mean with SEM corresponding to the above-mentioned time periods. (*) *p*<0.05, (**) p<0.01, (***) *p*<0.005, (§) 0.05<*p*<0.10. Further details in Methods.

Two predominant patterns of circulating cytokine levels were observed in aged mice: those increased with age regardless of parity -and then specifically altered in response to intraperitoneal tumor induction (Fig. 3), and those differing as a sole result of parity (manuscript in preparation). CCL2, IL10, IL5, IL4, TNF-α, IL1-beta and IL12p70 belong to the first pattern. TNF-α, IL1-β and IL4 were significantly increased in aged multiparous mice relative to young mice, and the same tendency was observed in the aged virgin group (TNF-α p=0.057; IL1-beta p=0.067; IL4 p=0.024, unpaired t-test with Welch’s correction). CXCL10 was distinctive in that no significant increase was observed with age but its parity-dependent tumor response was in the opposite direction with respect to the other cytokines. IL12p70 increased in both groups of aged mice relative to young mice, but their level variations upon tumor response did not reach significance.

### Cytokine profiles and host survival after intraperitoneal tumor induction

**Figure 4.**
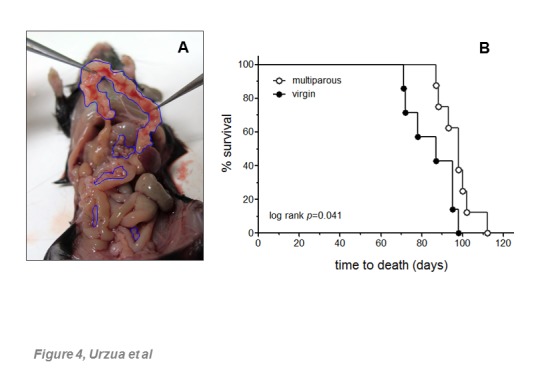
Tumor spread and survival of host aged mice **(A)** Demonstrative image of intraperitoneal tumor implants formed in a 19 months-old virgin C57BL6 female mouse injected with MOSE cells. Tumor implants in the omental fat band are shown pulled-out with clamps. **(B)** Survival plots of virgin and multiparous aged mice injected with IG-10 MOSE cells; day 0 corresponds to 16.1 ± 0.3 months of age for the two groups. Median survivals were 98 and 87 days for the multiparous and virgin groups, respectively. The *p* value of log-rank (Mantel-Cox) is shown. Both the log-rank and the Gehan-Breslow-Wilcoxon (*p*=0.038) tests were performed in GraphPad Prism 5 with 95% CI of 0.7638-1.489 for ratios of survival.

Spontaneously-transformed MOSE cells have been widely used as a syngeneic model of OC to study various disease aspects including intraperitoneal tumor dissemination and therapeutic approaches. Here, circulating cytokine levels in both virgin and multiparous mice during tumor progression are shown as dashed bars in Figure 3. As judged by abdominal enlargement in most cases, ascitic fluid started to accumulate 7-12 days before death, consistent with description provided by Roby *et al*., who developed this model in young adult mice [30]. Typically, abundant tumor implants were located in the omental fat band. Implants of smaller size were found in the diaphragm, mesenterium and the pelvic region next to ovaries/oviducts and uterine horns (Fig. 4A). Median survival times were 87 days (range 72-98) for virgin mice and 98 days (range 87-112) for multiparous mice. Survival curves were significantly different as determined by two tests (*p*=0.041, log-rank test; *p*=0.038, Gehan-Breslow-Wilcoxon Test). Figure 4B shows survival plots. The ascitic fluid volume, tumor load, and percentage of ascitic fluid with respect to body weight at time of death, were not statistically different between conditions.

## DISCUSSION

Natural ovarian decline is associated with hormone and inflammatory changes that overlap with those occurring as a consequence of aging in women. In addition, as suggested from epidemiological data in various populations, periods of suppressed ovulation plus P4 (or progestin) exposure over woman´s fertility span are major determinants of OC risk later in menopause [23]. We have approached this subject by using a syngeneic immunocompetent OC mouse model maintained under divergent host reproductive conditions (virgin and multiparous) until the peri-estropausal age. Using mouse to study age-associated cancer immunomodulation is supported by recent findings suggesting that the age-dependent evolution (adult, mature, old and long lived) of immune functions such as chemotaxis, phagocytosis, natural killer activity and lymphoproliferation is similar between humans and mice of equivalent chronological ages [32]. Estrous cycle lengthening and increased serum levels of the gonadotropins FSH and LH observed by ≥ 15 months-old in aged mice, were indicative of ovarian senescence and a declining control of the HPG axis. Since gonadotropins in aged animals were higher than in young, but lower than in ovariectomized mice (data not shown), mice of the age range studied in the present work would be equivalent to a human in late menopausal transition or early post-menopause [33].

Of interest was the significantly reduced PRL level in aged multiparous mice relative to aged virgin mice, a finding consistent with low PRL levels reported in parous women and in those taking oral contraceptives [34], factors known to decrease OC risk. PRL exerts pleiotropic metabolic and growth-like effects in various target tissues. In rodents, PRL is synthesized exclusively by the pituitary while in humans it is also produced by several tissues, most interestingly, immune B and T cells. The PRL profile during both the reproductive cycle and pregnancy differs between human and rodents. Besides its function in lactogenesis, PRL plays a luteotropic role in mice to maintain pregnancy [35]. Importantly, the first pregnancy in women induces a decrease of basal and stimulated PRL levels compared to nulliparous controls [36]. This effect has been replicated in rats and is linked to a decreased oxidative burst, enhanced phagocytic ability, as well as production of nitric oxide and TNF-α by peritoneal macrophages in multiparous animals [37, 38]. Increasing evidence involves elevated PRL levels in the pathogenesis of certain autoimmune diseases [39]. PRL has been recently proposed to impair the tumor-suppressive function of BRCA1 by downregulating expression of the cell cycle inhibitor p21 in cancer cell lines [40]. We also observed higher TSH levels in both aged mice groups regardless of parity history. Though normal serum TSH values have not been clearly defined in the elderly [41], TSH levels increase with age in humans [42] leading to an apparent sub-clinical hypothyroid condition which has been linked to the risk of atherosclerosis [43], among other chronic diseases.

From a total of 30 circulating cytokines measured in aged female mice in this study, 14 cytokines showed parity-dependent differential serum levels. Figure 3 shows a set of seven cytokines that increased with age, regardless of parity, and decreased in response to tumor induction only in multiparous mice. Notably, this pattern observed in multiparous animals was correlated with a significantly increased survival (Fig. 4B), suggesting that OC risk reduction associated with parity might involve a particular ability of the senescent immune system of parous mice to react better against the tumor challenge. An exception to this pattern was the chemokine CXCL10, which did not increase significantly with age but exhibited a unique and significant increase in multiparous mice upon tumor induction. A second major pattern included cytokines higher in intact multiparous mice as a sole result of parity (manuscript in preparation). As previously described in young adult mice [30], the MOSE cells used in this study induced ascitic fluid accumulation and extensive peritoneal carcinomatosis, mostly in the omental fat band of aged animals (Fig. 4A), both characteristics frequently observed in human OC [44].

Among these cytokines, we detected CCL2 (MCP-1) which participates in monocyte recruitment and tissue infiltration, with subsequent differentiation to macrophages. In agreement with the present results in mice, in women CCL2 increased during late menopausal transition showing a positive correlation with FSH [14]. CCL2 expression by luteal cells during *corpus luteum* (CL) regression enhances macrophage infiltration, a process that seems to be reversed by local P4 and luteotrophic prostaglandin E [45]. Only in multiparous aged mice, e.g., animals exposed to P4 during pregnancies early in life, did our results show lower CCL2 levels in response to tumor induction (Fig. 3). This tumor invasion-dependent decrease of CCL2 at estropause might represent a long-term effect of P4 on infiltration of tumor-associated macrophages (TAMs) during peritoneal tumor spread in this model. In agreement with our results, parity significantly reduced omental monocyte subsets and B1-B lymphocytes in the MOSE model at middle age, with concomitant decreased expression of various chemokines and polarization factors including CCL2 [46]. In other tissue contexts, recent reports indicate that progestin suppressed TNF-α induced proliferation and CCL2 secretion of endometrial stromal cells *in vitro* [47], while P4 prevented macrophage infiltration to brain endothelial cells by blocking CCL2 action after an ischemic stroke [48]. Importantly, high CCL2 levels have been reported in the ascitic fluid of OC patients [49].

In the present study, the Th2-class cytokines IL-10, IL-5 and IL4 were also decreased in response to the tumor challenge, but only in multiparous mice. Th1 and Th2 represent two CD4+ T-cells subsets each expressing a distinctive cytokine repertoire. Th1 cells are involved in cell-mediated immunity, whereas Th2 cells participate in the humoral immune response. The normal Th1/Th2 balance becomes altered with aging [50, 51] and during pregnancy [52]. Consistent with our results in aged mice, serum IL-10 and IL-4 increased at menopause [10, 13], whereas IL-5 has not yet been linked to women´s reproductive aging. Th1 and Th2 responses typically involve macrophage differentiation towards the M1 and M2 polarized phenotypes. The M2 shift observed in a subset of TAMs was characterized by high IL-10 expression leading to immunosuppression and subsequent tumor promotion [53]. Furthermore, OC stem cells promoted *in vitro* M2 polarization of raw 264.7 macrophages through the PPARγ/NF-κB pathway [54]. In our model, as the age-induced IL-10 increase was reversed only in multiparous mice exposed to tumorigenic cells, we suggest that parity counteracts tumor immunosuppression by partially repressing M2 polarization of TAMs.

Mediated by pregnancy hormones, the immune system undergoes adaptive changes to tolerate the fetus [55, 56]. Among these, mature B-cells accumulate in bone marrow, in lymph nodes draining the uterus and in the peritoneum of pregnant mice [57]. Both progesterone and estradiol repress IL-10 expression in a subpopulation of splenic B-cells during mouse pregnancy [58]. IL-10 is a major effector of B-cell immune regulatory properties [59], and has been recently implicated in the weak antitumor immunity observed in OC [60]. In contrast to the effect of IL-10 on B cells, a Th2 shift mediated by transient high IL-4 levels during initial stages of human pregnancy contributes to fetal tolerance and survival [52]. Interestingly, given that IL-4 receptors (IL-4R) are highly expressed in OC and other neoplasia, the IL-4/IL-4R pair has been attempted as a target for OC immunotherapy [61]. Because the age-dependent high levels of IL-10, IL-4 and IL-5 were subsequently decreased by tumor induction only in multiparous mice in the present model, we suggest that parity during fertile life might improve antitumor immunity in aged mouse challenged with tumor-inducing cells. In other words, in contrast to aged virgin mice which seem to be unresponsive to OC tumor invasion, the immune system of an aged multiparous mouse would be less tumor tolerant and able to better suppress OC growth. The question then becomes, which of the variables we have noted in multiparous versus virgin aged mice would be responsible for this protection? We have evidence for at least two converging mechanisms that could be in operation: impairment of the M2 macrophage shift and decreased stimulation of regulatory B-cells.

Similar to IL-10, the pro-inflammatory M1-type cytokines TNF-α and IL-1beta have both been shown to increase with age in women thus contributing to the systemic low-grade chronic inflammation status [11, 62]. TNF-α is produced mainly by activated macrophages, monocytes and lymphocytes, and participates in multiple normal physiological responses as well as pathogenic processes [63]. In the present study, the levels of TNF-α, IL1-β and IL12p70 increased in both aged groups irrespective of parity, and decreased as a response to tumor spread only in the parous group, in a similar fashion as observed for the M2-type cytokines (Fig. 3). Consistent with this, it was recently shown that a decreased level of TNF-α transcript is associated with parity in epithelial ovarian carcinomas [64]. Indeed, elevated TNF-α levels are associated to increased OC risk [16], while the combination of high TNF-α plus high IL-6 levels in ascitic fluid at primary surgery are predictors of rapid relapse in patients with epithelial OC [65]. Furthermore, the constitutive TNF-α expression by OC cells *in vitro* induces an autocrine network of additional cytokines including CCL2 [66]. Such links between TNF-α and CCL2 appear early during the fertility cycle in women, where TNF-α expression peaks in the regressing CL and correlates with CCL2 expression, despite the fact that it does not impair steroidogenesis in lutein-granulosa cells [45].

Monocytes, macrophages, dendritic cells and brain microglia are the major sources of IL1-β [67]. This cytokine plays diverse roles in reproductive ovarian function [68] and is found increased at post-menopause [9]. IL1-β promotes tumor invasion and angiogenesis while it depresses antitumor immunity [69]. As age-dependent increased IL1-β levels in the present study were reduced by tumor induction only in multiparous mice, we propose that long-term parity counteracts tumor spread by reducing angiogenesis and relieving immune suppression by the host tissue. Interestingly, FSH levels at pre-menopause were positively correlated with circulating IL-1β, IL-6 and TNF-α levels, while isolated mononuclear cells secreted these three cytokines upon exposure to exogenous FSH [5]. Given that FSH receptor expression has been also detected in osteoclasts [70], extragonadal tissue [71] and some genitourinary tumors [72], it seems feasible that the crosstalk between systemic FSH and cytokines including IL-1β, IL-6 and TNF-α might contribute to the pro-inflammatory status at post-menopause.

Finally, the pro-inflammatory cytokine IL-12p70 also followed the above described pattern. This is a M1-type heterodimeric cytokine composed of p35 and p40 kDa subunits coded by different genes. IL-12p70 is produced mostly by antigen-presenting cells such as macrophages and dendritic cells. It exerts an immune-stimulatory role on NK cells and promotes differentiation of naive CD4+ T lymphocytes into Th1 cells that produce IFN-γ and TNF-α as part of the innate and adaptive immune responses against endogenous damage [73]. Importantly, IL-12p70 was increased at menopause [11] and the level of p40 subunit was positively associated with parity [8]. As a potential cancer therapy, IL-12p70 reversed the immunosuppressive phenotype of tumor-associated macrophages in a subcutaneous murine model, with concomitant reduced synthesis of CCL2 and IL-10 [74]. In other work, reduction of omental tumors was observed in response to MOSE cells constitutively expressing membrane-bound IL-12p35p40 [75]. In both cases, IL-12p70 expression was effective only in a localized tumor microenvironment since its systemic administration caused toxicity [76]. IL-12p70 induced IFN-γ production, in turn, induces synthesis of the chemokine CXCL10 (IP-10; 10kD interferon γ-induced protein), which was the only cytokine that increased in multiparous mice in response to tumor induction. Thus, the divergent patterns of IL-12p70 and CXCL10 in our data appeared seemingly inconsistent. CXCL10 is chemotactic for monocytes and T-cells, and has both pro-apoptotic capacity and angiostatic properties against VEGF action, thereby acting as an antitumor agent [77].

An important aspect to consider in the present study, is that the levels of cytokines measured somehow reflect the near-senescent state of diverse tissues and organs of aged mice. Among various features, cellular senescence is characterized by secretion of a particular set of cytokines, chemokines, proteases and cell-matrix remodeling factors collectively known as the “senescence-associated secretory phenotype” (SASP) [78]. However, the relative contributions of each organ/tissue to the pool of circulating cytokines are difficult to estimate. Lastly, a further consequence of the SASP is that senescent tissues become infiltrated with immune cells. As an example, various morphological types of macrophages have been detected in the stroma of the aged mouse ovary in relation to follicle atresia, non-heme iron accumulation, presence of ceroid/lipofuscin and fibrosis [79, 80].

### Conclusions

Elevated serum gonadotropin levels and an extended length of estrous cycle indicated a peri-estropausal state in mice of both study groups >15 months old. PRL levels were lowest in multiparous mice, a finding also reported in multiparous women, and linked to decreased OC risk. Circulating levels of CCL2, IL10, IL5, IL4, TNF-α, IL1b and IL12p70 increased with age regardless of parity history, thus confirming a low-grade chronic inflammatory condition in all peri-estropausal female mice irrespective of parity. Importantly, the levels of these cytokines were significantly reduced in response to tumor induction only in the multiparous animals, suggesting that the drop in these cytokines levels was somehow associated with OC protection. CCL2, IL-10, IL-5 and IL-4 are typically implicated in M2 macrophage polarization, whereas TNF-α, IL1b and IL12p70 participate in M1 polarization. Similar to the human pattern, exogenous syngeneic tumorigenic MOSE cells formed tumor implants spreading across the peritoneal cavity predominantly in the *omentum*. Survival of multiparous mice was significantly longer than nulliparous ones, suggesting that parity would play a favorable prognostic role. We conclude that, in response to intraperitoneal tumor spread, multiparity partially reverts age-associated systemic inflammation while reducing immune-suppression against OC, *i.e*, past parity would improve the immune response against the tumor. A decrease of M2 polarization of tumor-associated macrophages, plus a reduced stimulation of regulatory B-cells might be important aspects of this effect, at least in the present mouse model. Further studies are needed to define how the studied cytokines modulate biological responses at the gene expression level in their multiple targets, and in senescent tissues as well as in tumor cells and their associated cellular microenvironments. To our knowledge, this is the first report describing a long-term effect of pregnancy on age-associated chronic inflammation relevant to OC in an animal model.

## References

[1] Franceschi C, Campisi J (2014). Chronic inflammation (inflammaging) and its potential contribution to age-associated diseases. J Gerontol A Biol Sci Med Sci, 69 Suppl 1: S4-9 24833586 10.1093/gerona/glu057

[2] Lopez-Otin C, Blasco MA, Partridge L, Serrano M, Kroemer G (2013). The hallmarks of aging. Cell, 153: 1194-1217 23746838 10.1016/j.cell.2013.05.039 PMC3836174

[3] Abu-Taha M, Rius C, Hermenegildo C, Noguera I, Cerda-Nicolas JM, Issekutz AC, et al (2009). Menopause and ovariectomy cause a low grade of systemic inflammation that may be prevented by chronic treatment with low doses of estrogen or losartan. J Immunol, 183: 1393-1402 19553526 10.4049/jimmunol.0803157

[4] Benedusi V, Meda C, Della Torre S, Monteleone G, Vegeto E, Maggi A (2012). A lack of ovarian function increases neuroinflammation in aged mice. Endocrinology, 153: 2777-2788 22492304 10.1210/en.2011-1925 PMC3359599

[5] Cannon JG, Cortez-Cooper M, Meaders E, Stallings J, Haddow S, Kraj B, et al (2010). Follicle-stimulating hormone, interleukin-1, and bone density in adult women. Am J Physiol Regul Integr Comp Physiol, 298: R790-798 20042686 10.1152/ajpregu.00728.2009 PMC2838653

[6] Lobo RA, Davis SR, De Villiers TJ, Gompel A, Henderson VW, Hodis HN, et al (2014). Prevention of diseases after menopause. Climacteric, 17: 540-556 24969415 10.3109/13697137.2014.933411

[7] Kim OY, Chae JS, Paik JK, Seo HS, Jang Y, Cavaillon JM, et al (2012). Effects of aging and menopause on serum interleukin-6 levels and peripheral blood mononuclear cell cytokine production in healthy nonobese women. Age (Dordr), 34: 415-425 21487705 10.1007/s11357-011-9244-2 PMC3312621

[8] Clendenen TV, Koenig KL, Arslan AA, Lukanova A, Berrino F, Gu Y, et al (2011). Factors associated with inflammation markers, a cross-sectional analysis. Cytokine, 56: 769-778 22015105 10.1016/j.cyto.2011.09.013 PMC3245985

[9] Malutan AM, Dan M, Nicolae C, Carmen M (2014). Proinflammatory and anti-inflammatory cytokine changes related to menopause. Prz Menopauzalny, 13: 162-168 26327849 10.5114/pm.2014.43818 PMC4520358

[10] Yasui T, Maegawa M, Tomita J, Miyatani Y, Yamada M, Uemura H, et al (2007). Changes in serum cytokine concentrations during the menopausal transition. Maturitas, 56: 396-403 17164077 10.1016/j.maturitas.2006.11.002

[11] Vural P, Canbaz M, Akgul C (2006). Effects of menopause and postmenopausal tibolone treatment on plasma TNFalpha, IL-4, IL-10, IL-12 cytokine pattern and some bone turnover markers. Pharmacol Res, 53: 367-371 16503406 10.1016/j.phrs.2006.01.005

[12] Cioffi M, Esposito K, Vietri MT, Gazzerro P, D’Auria A, Ardovino I, et al (2002). Cytokine pattern in postmenopause. Maturitas, 41: 187-192 11886764 10.1016/s0378-5122(01)00286-9

[13] Deguchi K, Kamada M, Irahara M, Maegawa M, Yamamoto S, Ohmoto Y, et al (2001). Postmenopausal changes in production of type 1 and type 2 cytokines and the effects of hormone replacement therapy. Menopause, 8: 266-273 11449084 10.1097/00042192-200107000-00008

[14] Tani A, Yasui T, Matsui S, Kato T, Kunimi K, Tsuchiya N, et al (2013). Different circulating levels of monocyte chemoattractant protein-1 and interleukin-8 during the menopausal transition. Cytokine, 62: 86-90 23490412 10.1016/j.cyto.2013.02.011

[15] Ose J, Schock H, Tjonneland A, Hansen L, Overvad K, Dossus L, et al (2015). Inflammatory Markers and Risk of Epithelial Ovarian Cancer by Tumor Subtypes: The EPIC Cohort. Cancer Epidemiol Biomarkers Prev, 24: 951-961 25855626 10.1158/1055-9965.EPI-14-1279-T PMC4454588

[16] Trabert B, Pinto L, Hartge P, Kemp T, Black A, Sherman ME, et al (2014). Pre-diagnostic serum levels of inflammation markers and risk of ovarian cancer in the prostate, lung, colorectal and ovarian cancer (PLCO) screening trial. Gynecol Oncol, 135: 297-304 25158036 10.1016/j.ygyno.2014.08.025 PMC4254357

[17] Charbonneau B, Goode EL, Kalli KR, Knutson KL, Derycke MS (2013). The immune system in the pathogenesis of ovarian cancer. Crit Rev Immunol, 33: 137-164 23582060 10.1615/critrevimmunol.2013006813 PMC3940260

[18] Aune G, Stunes AK, Lian AM, Reseland JE, Tingulstad S, Torp SH, et al (2012). Circulating interleukin-8 and plasminogen activator inhibitor-1 are increased in women with ovarian carcinoma. Results Immunol, 2: 190-195 24371583 10.1016/j.rinim.2012.10.003 PMC3862343

[19] Mertens-Walker I, Baxter RC, Marsh DJ (2012). Gonadotropin signalling in epithelial ovarian cancer. Cancer Lett, 324: 152-159 22634496 10.1016/j.canlet.2012.05.017

[20] Kass AS, Lea TE, Torjesen PA, Gulseth HC, Forre OT (2010). The association of luteinizing hormone and follicle-stimulating hormone with cytokines and markers of disease activity in rheumatoid arthritis: a case-control study. Scand J Rheumatol, 39: 109-117 20337546 10.3109/03009740903270607

[21] Tsilidis KK, Allen NE, Key TJ, Dossus L, Lukanova A, Bakken K, et al (2011). Oral contraceptive use and reproductive factors and risk of ovarian cancer in the European Prospective Investigation into Cancer and Nutrition. Br J Cancer, 105: 1436-1442 21915124 10.1038/bjc.2011.371 PMC3241548

[22] Bodelon C, Wentzensen N, Schonfeld SJ, Visvanathan K, Hartge P, Park Y, et al (2013). Hormonal risk factors and invasive epithelial ovarian cancer risk by parity. Br J Cancer, 109: 769-776 23820255 10.1038/bjc.2013.344 PMC3738139

[23] Hunn J, Rodriguez GC (2012). Ovarian cancer: etiology, risk factors, and epidemiology. Clin Obstet Gynecol, 55: 3-23 22343225 10.1097/GRF.0b013e31824b4611

[24] Murdoch WJ, Martinchick JF (2004). Oxidative damage to DNA of ovarian surface epithelial cells affected by ovulation: carcinogenic implication and chemoprevention. Exp Biol Med (Maywood), 229: 546-552 15169974 10.1177/153537020422900613

[25] Syed V, Ho SM (2003). Progesterone-induced apoptosis in immortalized normal and malignant human ovarian surface epithelial cells involves enhanced expression of FasL. Oncogene, 22: 6883-6890 14534535 10.1038/sj.onc.1206828

[26] Diep CH, Charles NJ, Gilks CB, Kalloger SE, Argenta PA, Lange CA (2013). Progesterone receptors induce FOXO1-dependent senescence in ovarian cancer cells. Cell Cycle, 12: 1433-1449 23574718 10.4161/cc.24550 PMC3674071

[27] Fleming JS, McQuillan HJ, Millier MJ, Beaugie CR, Livingstone V (2007). E-cadherin expression and bromodeoxyuridine incorporation during development of ovarian inclusion cysts in age-matched breeder and incessantly ovulated CD-1 mice. Reprod Biol Endocrinol, 5: 14 17425809 10.1186/1477-7827-5-14 PMC1855058

[28] Caligioni CS (2009). Assessing reproductive status/stages in mice. Curr Protoc Neurosci, Appendix 4: Appendix 4I 10.1002/0471142301.nsa04is48 PMC2755182 19575469

[29] Golde WT, Gollobin P, Rodriguez LL (2005). A rapid, simple, and humane method for submandibular bleeding of mice using a lancet. Lab Anim (NY), 34: 39-43 10.1038/laban1005-39 16195737

[30] Roby KF, Taylor CC, Sweetwood JP, Cheng Y, Pace JL, Tawfik O, et al (2000). Development of a syngeneic mouse model for events related to ovarian cancer. Carcinogenesis, 21: 585-591 10753190 10.1093/carcin/21.4.585

[31] Morton DB, Griffiths PH (1985). Guidelines on the recognition of pain, distress and discomfort in experimental animals and an hypothesis for assessment. Vet Rec, 116: 431-436 3923690 10.1136/vr.116.16.431

[32] Martinez de Toda I, Mate I, Vida C, Cruces J, De la Fuente M (2016). Immune function parameters as markers of biological age and predictors of longevity. Aging (Albany NY), 8: 3110-3119 27899767 10.18632/aging.101116 PMC5191888

[33] Finch CE (2014). The menopause and aging, a comparative perspective. J Steroid Biochem Mol Biol, 142: 132-141 23583565 10.1016/j.jsbmb.2013.03.010 PMC3773529

[34] Clendenen TV, Arslan AA, Lokshin AE, Liu M, Lundin E, Koenig KL, et al (2013). Circulating prolactin levels and risk of epithelial ovarian cancer. Cancer Causes Control, 24: 741-748 23378139 10.1007/s10552-013-0156-6 PMC3602319

[35] Ben-Jonathan N, LaPensee CR, LaPensee EW (2008). What can we learn from rodents about prolactin in humans? Endocr Rev, 29: 1-41 18057139 10.1210/er.2007-0017 PMC2244934

[36] Musey VC, Collins DC, Musey PI, Martino-Saltzman D, Preedy JR (1987). Long-term effect of a first pregnancy on the secretion of prolactin. N Engl J Med, 316: 229-234 3099198 10.1056/NEJM198701293160501

[37] Carvalho-Freitas MI, Anselmo-Franci JA, Palermo-Neto J, Felicio LF (2013). Prior reproductive experience alters prolactin-induced macrophage responses in pregnant rats. J Reprod Immunol, 99: 54-61 23714392 10.1016/j.jri.2013.03.005

[38] Tripathi A, Sodhi A (2007). Production of nitric oxide by murine peritoneal macrophages in vitro on treatment with prolactin and growth hormone: involvement of protein tyrosine kinases, Ca(++), and MAP kinase signal transduction pathways. Mol Immunol, 44: 3185-3194 17336385 10.1016/j.molimm.2007.01.024

[39] Shelly S, Boaz M, Orbach H (2012). Prolactin and autoimmunity. Autoimmun Rev, 11: A465-470 22155203 10.1016/j.autrev.2011.11.009

[40] Chen KH, Walker AM (2016). Prolactin inhibits a major tumor-suppressive function of wild type BRCA1. Cancer Lett, 375: 293-302 26970274 10.1016/j.canlet.2016.03.007

[41] Hennessey JV, Espaillat R (2015). Diagnosis and Management of Subclinical Hypothyroidism in Elderly Adults: A Review of the Literature. J Am Geriatr Soc, 63: 1663-1673 26200184 10.1111/jgs.13532

[42] Hollowell JG, Staehling NW, Flanders WD, Hannon WH, Gunter EW, Spencer CA, et al (2002). Serum TSH, T(4), and thyroid antibodies in the United States population (1988 to 1994): National Health and Nutrition Examination Survey (NHANES III). J Clin Endocrinol Metab, 87: 489-499 11836274 10.1210/jcem.87.2.8182

[43] Geng H, Zhang X, Wang C, Zhao M, Yu C, Zhang B, et al (2015). Even mildly elevated TSH is associated with an atherogenic lipid profile in postmenopausal women with subclinical hypothyroidism. Endocr Res, 40: 1-7 24679183 10.3109/07435800.2013.879166

[44] Thibault B, Castells M, Delord JP, Couderc B (2014). Ovarian cancer microenvironment: implications for cancer dissemination and chemoresistance acquisition. Cancer Metastasis Rev, 33: 17-39 24357056 10.1007/s10555-013-9456-2

[45] Nio-Kobayashi J, Kudo M, Sakuragi N, Kimura S, Iwanaga T, Duncan WC (2015). Regulated C-C motif ligand 2 (CCL2) in luteal cells contributes to macrophage infiltration into the human corpus luteum during luteolysis. Mol Hum Reprod, 21: 645-654 26003810 10.1093/molehr/gav028

[46] Cohen CA, Shea AA, Heffron CL, Schmelz EM, Roberts PC (2013). The parity-associated microenvironmental niche in the omental fat band is refractory to ovarian cancer metastasis. Cancer Prev Res (Phila), 6: 1182-1193 24022590 10.1158/1940-6207.CAPR-13-0227 PMC3836366

[47] Grandi G, Mueller M, Bersinger N, Papadia A, Nirgianakis K, Cagnacci A, et al (2016). Progestin suppressed inflammation and cell viability of tumor necrosis factor-alpha-stimulated endometriotic stromal cells. Am J Reprod Immunol, 76: 292-298 27515307 10.1111/aji.12552

[48] Remus EW, Sayeed I, Won S, Lyle AN, Stein DG (2015). Progesterone protects endothelial cells after cerebrovascular occlusion by decreasing MCP-1- and CXCL1-mediated macrophage infiltration. Exp Neurol, 271: 401-408 26188381 10.1016/j.expneurol.2015.07.010 PMC4586408

[49] Mikula-Pietrasik J, Uruski P, Szubert S, Moszynski R, Szpurek D, Sajdak S, et al (2016). Biochemical composition of malignant ascites determines high aggressiveness of undifferentiated ovarian tumors. Med Oncol, 33: 94 27431203 10.1007/s12032-016-0810-4

[50] Mahbub S, Deburghgraeve CR, Kovacs EJ (2012). Advanced age impairs macrophage polarization. J Interferon Cytokine Res, 32: 18-26 22175541 10.1089/jir.2011.0058 PMC3255514

[51] Mansfield AS, Nevala WK, Dronca RS, Leontovich AA, Shuster L, Markovic SN (2012). Normal aging is associated with an increase in Th2 cells, MCP-1 (CCL1) and RANTES (CCL5), with differences in sCD40L and PDGF-AA between sexes. Clin Exp Immunol, 170: 186-193 23039889 10.1111/j.1365-2249.2012.04644.x PMC3482365

[52] Challis JR, Lockwood CJ, Myatt L, Norman JE, Strauss JF 3rd, Petraglia F (2009). Inflammation and pregnancy. Reprod Sci, 16: 206-215 19208789 10.1177/1933719108329095

[53] Sica A, Mantovani A (2012). Macrophage plasticity and polarization: in vivo veritas. J Clin Invest, 122: 787-795 22378047 10.1172/JCI59643 PMC3287223

[54] Deng X, Zhang P, Liang T, Deng S, Chen X, Zhu L (2015). Ovarian cancer stem cells induce the M2 polarization of macrophages through the PPARgamma and NF-kappaB pathways. Int J Mol Med, 36: 449-454 26035689 10.3892/ijmm.2015.2230

[55] Schumacher A, Costa SD, Zenclussen AC (2014). Endocrine factors modulating immune responses in pregnancy. Front Immunol, 5: 196 24847324 10.3389/fimmu.2014.00196 PMC4021116

[56] Lissauer D, Eldershaw SA, Inman CF, Coomarasamy A, Moss PA, Kilby MD (2015). Progesterone promotes maternal-fetal tolerance by reducing human maternal T-cell polyfunctionality and inducing a specific cytokine profile. Eur J Immunol, 45: 2858-2872 26249148 10.1002/eji.201445404 PMC4833190

[57] Muzzio DO, Soldati R, Ehrhardt J, Utpatel K, Evert M, Zenclussen AC, et al (2014). B cell development undergoes profound modifications and adaptations during pregnancy in mice. Biol Reprod, 91: 115 25210132 10.1095/biolreprod.114.122366

[58] Bommer I, Muzzio DO, Zygmunt M, Jensen F (2016). Progesterone and estradiol exert an inhibitory effect on the production of anti-inflammatory cytokine IL-10 by activated MZ B cells. J Reprod Immunol, 116: 113-116 27317920 10.1016/j.jri.2016.05.008

[59] Schwartz M, Zhang Y, Rosenblatt JD (2016). B cell regulation of the anti-tumor response and role in carcinogenesis. J Immunother Cancer, 4: 40 27437104 10.1186/s40425-016-0145-x PMC4950763

[60] Wei X, Jin Y, Tian Y, Zhang H, Wu J, Lu W, et al (2016). Regulatory B cells contribute to the impaired antitumor immunity in ovarian cancer patients. Tumour Biol, 37: 6581-6588 26638169 10.1007/s13277-015-4538-0

[61] Kioi M, Takahashi S, Kawakami M, Kawakami K, Kreitman RJ, Puri RK (2005). Expression and targeting of interleukin-4 receptor for primary and advanced ovarian cancer therapy. Cancer Res, 65: 8388-8396 16166317 10.1158/0008-5472.CAN-05-1043

[62] Michaud M, Balardy L, Moulis G, Gaudin C, Peyrot C, Vellas B, et al (2013). Proinflammatory cytokines, aging, and age-related diseases. J Am Med Dir Assoc, 14: 877-882 23792036 10.1016/j.jamda.2013.05.009

[63] Bradley JR (2008). TNF-mediated inflammatory disease. J Pathol, 214: 149-160 18161752 10.1002/path.2287

[64] Gupta M, Babic A, Beck AH, Terry K (2016). TNF-alpha expression, risk factors, and inflammatory exposures in ovarian cancer: evidence for an inflammatory pathway of ovarian carcinogenesis? Hum Pathol, 54: 82-91 27068525 10.1016/j.humpath.2016.03.006 PMC4938714

[65] Kolomeyevskaya N, Eng KH, Khan AN, Grzankowski KS, Singel KL, Moysich K, et al (2015). Cytokine profiling of ascites at primary surgery identifies an interaction of tumor necrosis factor-alpha and interleukin-6 in predicting reduced progression-free survival in epithelial ovarian cancer. Gynecol Oncol, 138: 352-357 26001328 10.1016/j.ygyno.2015.05.009 PMC4522366

[66] Kulbe H, Thompson R, Wilson JL, Robinson S, Hagemann T, Fatah R, et al (2007). The inflammatory cytokine tumor necrosis factor-alpha generates an autocrine tumor-promoting network in epithelial ovarian cancer cells. Cancer Res, 67: 585-592 17234767 10.1158/0008-5472.CAN-06-2941 PMC2679985

[67] Garlanda C, Dinarello CA, Mantovani A (2013). The interleukin-1 family: back to the future. Immunity, 39: 1003-1018 24332029 10.1016/j.immuni.2013.11.010 PMC3933951

[68] Gerard N, Caillaud M, Martoriati A, Goudet G, Lalmanach AC (2004). The interleukin-1 system and female reproduction. J Endocrinol, 180: 203-212 14765973 10.1677/joe.0.1800203

[69] Song X, Voronov E, Dvorkin T, Fima E, Cagnano E, Benharroch D, et al (2003). Differential effects of IL-1 alpha and IL-1 beta on tumorigenicity patterns and invasiveness. J Immunol, 171: 6448-6456 14662844 10.4049/jimmunol.171.12.6448

[70] Sun L, Peng Y, Sharrow AC, Iqbal J, Zhang Z, Papachristou DJ, et al (2006). FSH directly regulates bone mass. Cell, 125: 247-260 16630814 10.1016/j.cell.2006.01.051

[71] Stilley JA, Christensen DE, Dahlem KB, Guan R, Santillan DA, England SK, et al (2014). FSH receptor (FSHR) expression in human extragonadal reproductive tissues and the developing placenta, and the impact of its deletion on pregnancy in mice. Biol Reprod, 91: 74 25100706 10.1095/biolreprod.114.118562 PMC4435062

[72] Gartrell BA, Tsao CK, Galsky MD (2013). The follicle-stimulating hormone receptor: a novel target in genitourinary malignancies. Urol Oncol, 31: 1403-1407 22513137 10.1016/j.urolonc.2012.03.005 PMC4744483

[73] Zheng H, Ban Y, Wei F, Ma X (2016). Regulation of Interleukin-12 Production in Antigen-Presenting Cells. Adv Exp Med Biol, 941: 117-138 27734411 10.1007/978-94-024-0921-5_6

[74] Watkins SK, Egilmez NK, Suttles J, Stout RD (2007). IL-12 rapidly alters the functional profile of tumor-associated and tumor-infiltrating macrophages in vitro and in vivo. J Immunol, 178: 1357-1362 17237382 10.4049/jimmunol.178.3.1357

[75] Cohen CA, Shea AA, Heffron CL, Schmelz EM, Roberts PC (2016). Interleukin-12 Immunomodulation Delays the Onset of Lethal Peritoneal Disease of Ovarian Cancer. J Interferon Cytokine Res, 36: 62-73 26430781 10.1089/jir.2015.0049 PMC4722576

[76] Hernandez-Alcoceba R, Poutou J, Ballesteros-Briones MC, Smerdou C (2016). Gene therapy approaches against cancer using in vivo and ex vivo gene transfer of interleukin-12. Immunotherapy, 8: 179-198 26786809 10.2217/imt.15.109

[77] Liu M, Guo S, Stiles JK (2011). The emerging role of CXCL10 in cancer (Review). Oncol Lett, 2: 583-589 22848232 10.3892/ol.2011.300 PMC3406435

[78] Campisi J, Andersen JK, Kapahi P, Melov S (2011). Cellular senescence: a link between cancer and age-related degenerative disease? Semin Cancer Biol, 21: 354-359 21925603 10.1016/j.semcancer.2011.09.001 PMC3230665

[79] Asano Y (2012). Age-related accumulation of non-heme ferric and ferrous iron in mouse ovarian stroma visualized by sensitive non-heme iron histochemistry. J Histochem Cytochem, 60: 229-242 22108647 10.1369/0022155411431734 PMC3351130

[80] Briley SM, Jasti S, McCracken JM, Hornick JE, Fegley B, Pritchard MT, et al (2016). Reproductive age-associated fibrosis in the stroma of the mammalian ovary. Reproduction, 152: 245-260 27491879 10.1530/REP-16-0129 PMC4979755

